# Acquired right-sided diaphragmatic hernia after pediatric living donor transplantation: a guide for the non-transplant pediatric surgeon

**DOI:** 10.3389/fped.2025.1525423

**Published:** 2025-06-06

**Authors:** Francesca Palmisani, Hannah N. Stundner-Ladenhauf, Judith Pichler, Denise Aldrian, Andreas Heilos, Philipp Steinbauer, Martin L. Metzelder, Janina M. Patsch, Wolf-Dieter Huber, Rupert Oberhuber, Wilfried Krois

**Affiliations:** ^1^ Department of Pediatric Surgery, Medical University of Vienna, Vienna, Austria; ^2^ Department of Visceral, Transplant and Thoracic Surgery, Medical University of Innsbruck, Innsbruck, Austria; ^3^ Department of Pediatric Nephrology and Gastroenterology, Medical University of Vienna, Vienna, Austria; ^4^ Department of Paediatrics I, Medical University of Innsbruck, Innsbruck, Austria; ^5^ Department of Biomedical Imaging and Image-Guided Therapy, Medical University of Vienna, Vienna, Austria

**Keywords:** acquired diaphragmatic hernia, pediatric surgery, pediatric living-donor liver transplantation (pLDLT), multicenter study, split-liver transplantation, centralization

## Abstract

**Background:**

Pediatric living donor liver transplantation (pLDLT) has risen to standard of care for children with liver failure. Strong policies of centralization have led to exceptional results, reducing the risk of morbidity and mortality. As a counterpart, long term follow-up often occurs in centers with no dedicated transplant unit, that should familiarize themselves with the exceptional anatomy of the liver-transplanted child and their unique postoperative complications.

**Methods:**

We reviewed all cases of acquired diaphragmatic hernia (DH) after pLDLT in the period following the establishment of a strong policy of centralization in Austria, between January 2017 and May 2023. All patients were referred for liver transplantation to the newly established national transplant reference center (Medical University of Innsbruck, Austria). Postoperative follow up was conducted either at the transplant center or at their home institutions.

**Results:**

Of the 42 patients which received a pLDLT in the national reference center during the study period, 3 developed an acquired diaphragmatic hernia within the first eight months postoperatively (7%). All patients required emergent surgical treatment in a non-transplant center. All the cases presented with a defect in the posteromedial aspect of the diaphragm, potentially related to thermal effects in the bare area of the diaphragm during transplantation.

**Conclusions:**

Acquired diaphragmatic hernia is a rare complication of pLDLT, that mostly occurs in the long-term postoperative follow-up. Accurate knowledge of the surgical site is crucial to assure assessment and management in absence of the transplant-team. With this retrospective analysis we aim to enhance focus on post-liver-transplant complications and offer a guide for the non-transplant pediatric surgeon to raise awareness to post-operative anatomical alterations in these patients.

## Highlights

1. **What is currently known about this topic?**

Acquired right-sided diaphragmatic hernia after pediatric liver transplantation is a rare condition which has been described in sparse case reports mostly directed to the attention of transplant surgeons.

2. **What new information is contained in this article**

As centralization policies have been established in most countries to assure better outcomes of the pediatric transplants, long-term follow-up often occurs in centers with no transplant-units. After reporting our national experience, we aim to offer a guide to the non-transplant pediatric surgeons who need to face the acute management in areas where immediate transfer to a specialized pediatric transplant-center is limited.

## Introduction

Since the first successful procedure in 1989, split-liver living donor transplantation has become the gold standard of treatment for children with end-stage liver disease, with excellent outcomes (1). In the past three decades pediatric liver transplantation has indeed evolved remarkably, achieving survival rates over 85% and decreasing the waiting-list mortality rate to less than 10% (2). This has been made possible through refinements in surgical techniques aimed at maximizing donor organ use, such as reduced-size-liver transplantation and split-liver transplantation, which further gave way to living donor transplantation (1, 3).

Despite initial concerns regarding the safety of such practices, current literature actually shows better outcomes with living-donor liver transplantation (LDLT) as opposed to deceased donor grafts (4).

A critical role in enabling these successful outcomes is due to the strong regulations and policies of centralization, which have led to the establishment of a single pediatric transplant center in smaller countries like Austria. As a natural consequence, there is a growing cohort of children after pediatric living-donor liver transplantation (pLDLT), who are currently receiving long-term follow-up in centers without a dedicated pediatric transplant unit. In these cases, management of acute and long-term complications is often in the hands of non-transplant pediatric surgeons. Correspondingly, pediatric surgeons should familiarize themselves with the complex post-surgical anatomy of the liver transplanted child.

The aim of this paper is to retrospectively review all cases of acquired diaphragmatic hernia after pLDLT which have been registered in Austria after the centralization process. Long term follow up and treatment of acute complications mostly takes place in home institutions and expedite transfer of patients to a specialized transplant unit is often not possible due to geographical distance.

Through the consideration of patho-anatomy and patho-physiology of liver-transplanted children, our intention is to assist other non-transplanting pediatric surgeons in the assessment and acute management of such patients, in areas where immediate transfer to a specialized pediatric transplant-center is limited.

## Methods

We retrospectively reviewed the electronic records of acute or elective surgery for acquired diaphragmatic hernia (DH) after pLDLT of all patients who received a living donor liver transplantation in the national transplant center (Medical University of Innsbruck) in the period between January 2017 and May 2023. All patients under the age of 18 were included. Data regarding primary disease, eventual associated conditions, pre-transplantation status, intraoperative transplant characteristics and postoperative course has been evaluated.

This time frame was chosen due to an implementation of a strong centralization policy in Austria in 2017. Starting from 2017 all pediatric patients warranting liver transplantation were referred for liver transplantation to the newly established national transplant reference center (Medical University of Innsbruck, Austria).

The total number of pLDLT in the study period was retrieved from the transplantation yearly reports of Eurotransplant (5).

## Results

During our study period a total of 67 pediatric liver transplants have been performed at the Medical University of Innsbruck, of these 51 were split-liver transplants, with 42 being from living donors.

Three cases of acquired diaphragmatic hernia were found (2 males, 1 female, mean age of 3 years), all within eight months after liver transplantation. One case presented with a complex postoperative course of two acute recurrences of the diaphragmatic hernia at eight and eleven months from the initial repair. In all cases a pLDLT had been performed in the Medical University of Innsbruck. All emergency DH repair have been performed at the Medical University of Vienna.

Complete perioperative characteristics of the patients are depicted in Table 1. All three cases presented with acute symptoms of an incarcerated hernia and could not be transferred to the transplant center. Their clinical presentation differed due to the underlying variance in incarcerated content and site of obstruction. The first patient presented with symptoms of an acute intestinal obstruction with bilious vomiting and no passage of stool. In the second patient, the intestinal passage was not affected, but signs of cholestasis and biliodigestive obstruction with acute onset of elevated liver enzymes, jaundice, and change of stool-color to acholic were leading signs. The third patient presented with two acute recurrences of an initially asymptomatic diaphragmatic hernia, both times with signs of intestinal obstruction as well as cardio-pulmonary distress. In all cases the diaphragmatic defect was located at the right posteromedial paracaval area of the diaphragm.

**Table 1:** Patients demographics, perioperative characteristics.

Gender	Male	Male	Female
Primary disease	Acute liver failure (unknown cause)	Hepatoblastoma IV grade	Intrahepatic cholestasis, fibrosis
Associated disease	None	Lung metastasis, hypereosinophilia, autism	Multisystemic Langerhanscellhistiocytosis, BRAF mutation
Previous operations	None	Implantation of Hickman catheter thoracotomy and wedge resection for pulmonary metastasis	Implantation Hickman catheter
Condition before transplant			
Malnutrition	None	No	No
Hospitalization, medical treatment		chemotherapy accoding to PHITT protocol	chemotherapy LCH-IV protocol (vinblastin, prednisolon)
Renal impairment		No	No
Ascitic decompensation	Yes	Yes	No
Transplantation			
Age at tx (mo)	51	29	25
Weight at tx (kg)	17,5	12.5	9.4
Donor weight (kg)	82	61	64
Z score	0.24	−0.16	−3.79
Graft type	Segment II/III	Segment II/III	Segment II/III
Ischemic time (min)	00:49	00:23	00:58
Operative time	07:29	05:52	06:38
Abdominal closure	Primary	Primary	Primary
Post-TX course			
Complications	Ascites and pleural effusion	Acute bleeding and hematoma at liver hilus	Hepatic artery thrombosis, portal vein stenosis, portal hypertension, pleural effusion
Reoperation	Drainage	Relaparaotomy (pod 15)	Relaparotomy, arterial revision (pod 18), pleural drainage

### Case 1

**Figure 1:**
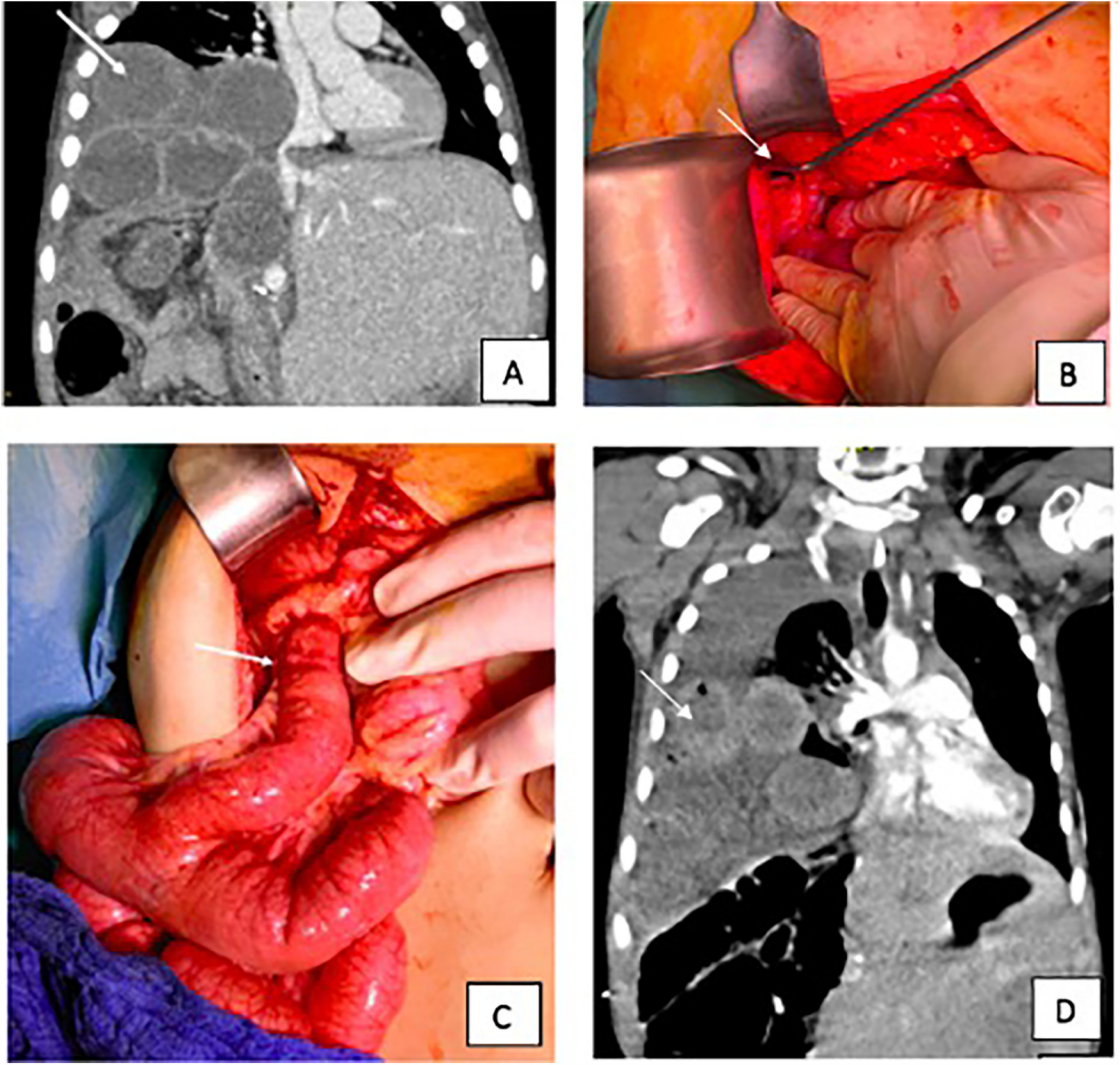
**(A)** Coronal view of CT image (with iodinated contrast); the arrows point at the herniated bowel loops (patient 1) **(B)** diaphragmatic defect (patient 1); the arrow points at the defect. **(C)** Herniated Roux-en-Y bilioenteric loop in the breached diaphragm (Patient 2); the arrow points at the herniated loop. **(D)** Coronal view of CT image (with iodinated contrast) at the time of first recurrence (Patient 3); the arrow points at the herniated bowel in the thoracic cavity.

A 4-year-old boy presented at his primary center of care with acute onset of vomiting, abdominal pain and distension two months after pLDLT due to pediatric acute liver failure (PALF) of unknown cause. Thoraco-abdominal computed tomography (CT) revealed the presence of an incarcerated right-sided diaphragmatic defect with compression of the inferior vena cava as well as free air (Figure 1A). Laparotomy was performed along the incision of the transplant scar. Intraoperative findings were of an incarcerated Roux-en-Y hepaticojejunostomy right paramedian to the vena cava (Figure 1B). To release and reduce the intrathoracic incarcerated loops, the diaphragmatic defect had to be extended about 2 cm laterally to facilitate relocation and in order to prevent perforation of bowel loops. Due to persistent ischemia a 10 cm resection with primary anastomosis was finally considered inevitable. The diaphragmatic defect was closed with interrupted monofilament 2–0 non-resorbable sutures without tension.

The postoperative course was uneventful, and the child was discharged 14 days postoperatively, fully tolerating oral feeds and with normal liver function parameters.

### Case 2

A 3-year-old boy presented to his primary center of care with recurrent vomiting and acholic stools six months after pLDLT due to metastatic hepatoblastoma. Elevation of inflammatory markers and liver function parameters were detected. Imaging revealed the presence of a transdiaphragmatic herniation of the Roux-en-Y hepaticojejunostomy, with small-bowel dilation and partial compression of the vena cava. Intraoperative findings were concordant with radiological imaging (Figure 1C). Extension of the transverse transplant scar to the midline up the xiphoid was necessary to achieve a sufficient operative view. A diaphragmatic defect of approximately 2 cm diameter was found at the right posteromedial paracaval area. The herniated bilio-enteric anastomosis was reduced into the abdomen and the diaphragmatic defect was closed with interrupted monofilament 2-0 non-resorbable sutures without tension. No perforation or ischemic bowel was noted. The postoperative course was uneventful, with the exception of a COVID-19 infection, which required the patient to be transferred to a dedicated COVID-19 unit. Liver function parameters were found to be within normal range by the 5th postoperative day. Discharge was uneventful 15 days postoperatively.

### Case 3

A 2-year-old girl was diagnosed with an asymptomatic diaphragmatic hernia on routine postoperative ultrasound six months after pLDLT performed due to liver failure in the context of systemic Langerhans cell histiocytosis. The patient was referred to her transplant center, where a 2.7 cm diaphragmatic defect was revealed on magnetic resonance imaging (MRI), with no signs of herniation of the liver (Figure 1D). Surgical correction of the defect was scheduled as an elective procedure and performed using a Gore-Tex patch and interrupted non-resorbable 2-0 sutures*.* There were no complications postoperatively and the patient was discharged on postoperative day 7.

Eight months later, the patient presented with acute vomiting and respiratory distress at her home insitution. Imaging revealed recurrence of the diaphragmatic defect with incarcerated bowel loops, causing mediastinal shift and pleural effusion. Due to her unstable condition, emergency surgery was performed locally. Intraoperative findings showed partial failure of the previously implanted Gore Tex-patch. The defect was closed primarily with interrupted sutures after reducing the bowel loops, which showed diffuse ischemia but recovered fully 48 h later. Postoperatively, complications included a recurrence of Langerhans cell histiocytosis and adhesive small bowel obstruction, requiring further emergency surgery. Subsequently, the patient experienced recurrent abdominal issues, leading to another diaphragmatic hernia recurrence, which was addressed thoracoscopically. No further recurrences of the hernia have been observed, though unrelated abdominal surgeries were necessary.

## Discussion

Despite the indisputable advances of surgical techniques and donor availability due to living donor liver transplantation over the past years, abdominal postoperative complications are frequent and result in high reoperation rates of approximately 30% (6). Most common complications include biliary strictures, biliary leaks, vascular thrombosis or hemorrhage, which most often affect the acute postoperative period and can usually be managed by the transplant team as the patient is still under their care. However, long-term complications such as diaphragmatic defects, bowel perforation and bowel- or biliary obstruction may occur after referral of the patient to their local center of care (7, 8).

The incidence of postoperative intestinal complications has indeed been reported to range up to 20%, with a high mortality rate (9).

Especially in case of intestinal obstruction, the surgeon on call needs to be aware that it may be caused by unique circumstances (6–8), as observed in our three patients.

Firstly, it needs to be noted that whilst intestinal obstruction is a common complication following any kind of abdominal surgery, its occurrence after pediatric liver transplantation is rare and has a reported incidence of 3.8% (7). Possible causes of intestinal obstruction in these patients include ventral and internal herniation, intussuseption, volvulus as well as post-transplantation lymphoproliferative disease (6–8).

A recent review on intestinal complications in post-transplant children has highlighted a few possible risk factors in the occurrence of bowel obstruction. These include low body mass index (BMI), previous abdominal surgeries, especially Kasai portoenterostomy, as well as longer operating times during the transplant (9). These findings were however not reflected by our population, as only one patient was under the first percentile for weight (and with a Z score <−2), none had biliary atresia as underlying disease, and none underwent any previous abdominal surgeries. Operating times were in line with the center's experience in all of the three cases.

Acquired diaphragmatic hernia should be considered as a differential diagnosis in children with signs of intestinal obstruction or respiratory distress following split liver transplantation, and knowledge of the post-transplant anatomy for non-transplanting pediatric surgeons is crucial to avoid further complications.

To enhance knowledge of the distinct circumstances in these patients, we aim to give an outlook into the basic anatomical and surgical principles of pLDLT.

### Anatomic and surgical principles of pediatric liver transplantation

Because most pediatric patients are very young (under the age of one) at the time of listing, the number of available organs and graft size mismatch are a constant struggle. While only a limited number of pediatric organs are available, correct graft size is difficult to achieve, even though it is a crucial prognostic factor for the success of the procedure. A small-for-size graft bears an increased risk of dysfunction, whilst large-for-size grafts may suffer from inadequate perfusion, thus incurring possible vascular complications (10). The latter may furthermore increase the risk of compartment syndrome, hampering the possibilities of a primary abdominal closure (11). According to recent studies, the correct graft-to-recipient weight ratio for optimal outcome should be between 0.6 and 1.0 (12).

Therefore, more and more patients are offered a split-liver transplant either from living or deceased donors. The technique of a split-liver transplant exploits the unique characteristic of the liver to be made of self-contained units, each with its independent vascular and biliary system. Accurate resection of a segment including its arterial and portal inflow as well as its venous and biliary drainage therefore allows individual transplantation (3). Based on their size, children usually receive segments 2 and 3 (left lateral segment, LLS), segments 2,3 and 4 (left lobe, LL) or rarely monosegments (segment 2 or segment 3) (2, 3, 12–14).

The primary surgical step in the recipient split liver transplantation is the hepatectomy, which is performed through a bilateral subcostal incision, possibly extended to the xiphoid. The hepatectomy is performed as close to the liver hilum as possible, to provide sufficient length of the hepatic artery, portal venous structures and common hepatic duct for the following anastomosis. All diaphragmatic attachments to the liver need to be dissected for adequate mobilization, step that in our opinion may be at the base of the described complication. After mobilization of the liver from the infrahepatic vena cava and finalization of the back-table preparation of the donor-organ, the portal vein and hepatic veins are individually clamped and the liver is then removed. Depending on the graft type, the retrohepatic vena cava can either be preserved (piggy-back-technique) or removed with the native liver (conventional method).

Transplantation of the donor organ occurs with venous, portal and arterial anastomosis. The biliary reconstruction is achieved by hepaticojejunostomy (2, 3, 11).

## Incarcerated acquired diaphragmatic hernia

**Figure 2:**
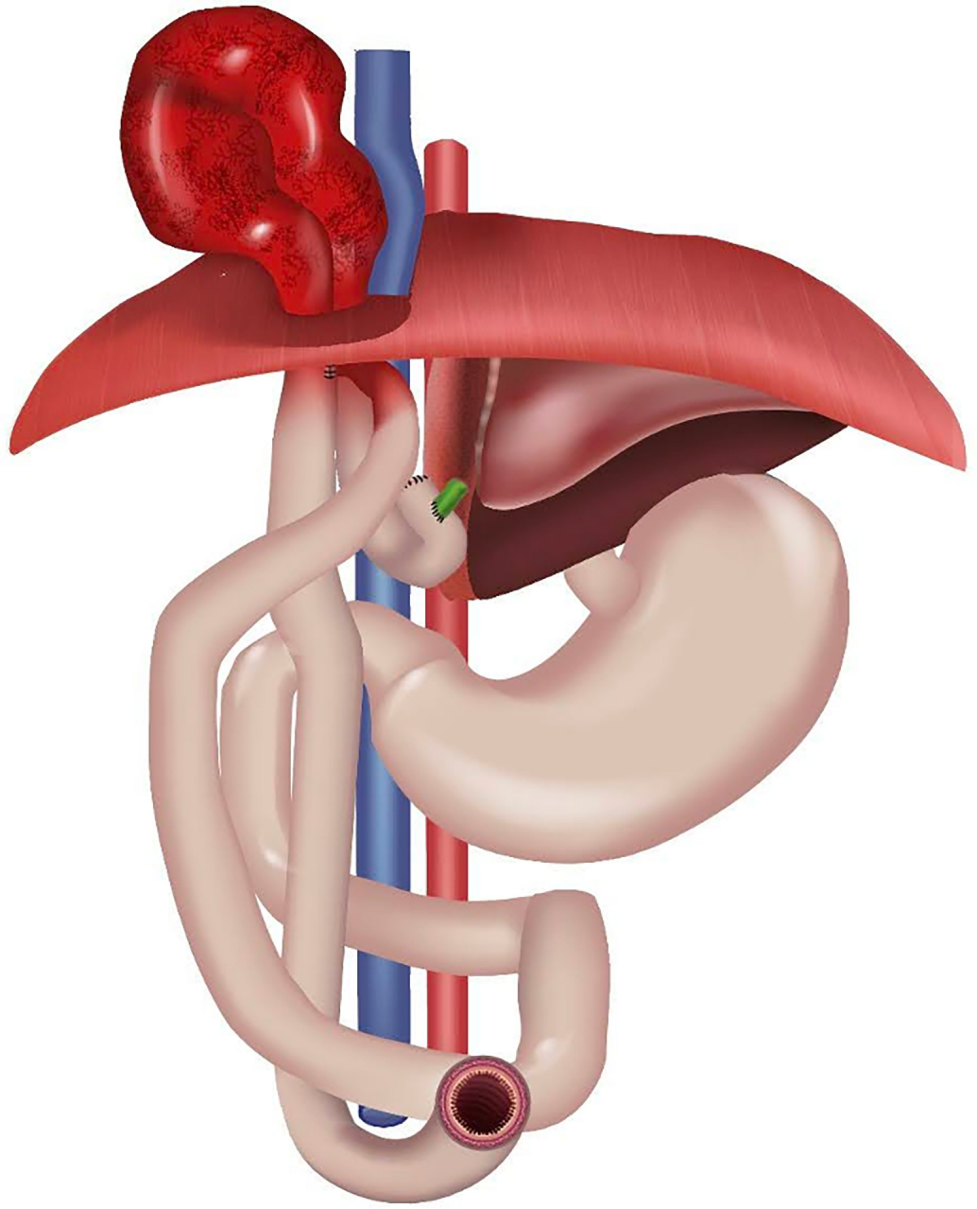
Schematic anatomy of DH after pLDLT.

Acquired iatrogenic diaphragmatic hernia (DH) is a rare complication and possible cause of intestinal obstruction after pLDLT (7, 8, 11, 15, 16). Figure 2 shows a schematic anatomy.

As investigated by Martin et al., the incidence of acquired diaphragmatic hernia seems to be especially increased in pediatric patients undergoing LLS transplantation in comparison to adult transplant recipients (17). According to recent literature, entirely based on case reports or limited case series, it has a reported incidence of 1.5%–2.8% (16, 18). It has to be noted, that incidence is generally reported with respect to all pediatric liver transplantation of high volume centers, without restriction to pLDLT. This may partially explain the higher incidence in our data evaluation.

Incarceration of diaphragmatic hernias have only been sparsely reported (18–20), and only in three cases the Roux-en-Y biliodigestive anastomosis was involved (18, 20).

Symptoms of DH may not be limited to bowel obstruction and may include respiratory symptoms (16).

It has been speculated that predisposing factors for DH in pediatric liver transplant patients may include extensive use of diathermy on the diaphragmatic surface, hindering the blood supply to the diaphragm. It is known that the most sensible anatomic areas of the diaphragm are the lumbocostal triangle, posterolaterally, and the so called “bare area”, posteromedially, which corresponds to the attachment area of the liver to the diaphragm and which lacks peritoneal coverage. In the latter, the presence of collateral vessels, which may require extensive coagulation during transplantation, may be a precipitating cause of damage to the integrity of the diaphragmatic tissue (16, 21).

Since all our cases presented with a defect in the posteromedial aspect of the diaphragm, in contrast with the majority of the cases reported so far (15, 22), we believe that indeed diathermic injury at the bare area may impair the structure of the diaphragm and therefore facilitate the occurrence of a diaphragmatic hernia. It seems that segmental allografts (LLS, left lateral segment) may increase the risk of herniation, as they leave a larger surface of the diaphragm uncovered. According to the literature, this would allow exposure of the diaphragm to the pressure of adjacent bowel loops, increasing the risk of DH (23). Furthermore, increased intra-abdominal pressure due to ascites, secondary abdominal closure or large-for-size grafts may also play a precipitating role in the development of the diaphragmatic breach.

This needs to be seen in close context with the overall conditions of the transplanted child, in which immunosuppression, malnutrition due to chronic liver disease and sarcopenia may contribute to diaphragmatic vulnerability (7, 8, 15, 18, 20, 24). Indeed, the use of steroids and mTOR inhibitors such as Sirolimus are known to delay wound healing, with the latter being reported to cause spontaneous rupture of the diaphragm (25). However, none of our children received steroids or were on mTOR inhibitors.

It is plausible that multiple factors contribute to the occurrence of an acquired diaphragmatic defect as described in our patients. All patients presented after a complicated postoperative course, which required re-interventions (drainage in one case, re-laparotomy in two). This may possibly lead to an increased intra-abdominal pressure in the early postoperative period, predisposing the patients to a diaphragmatic defect.

As all three cases had undergone liver transplantation with a LLS graft, we believe—as in accordance with the current literature (23)—that an LLS graft increases the risk of postoperative diaphragmatic defects. This knowledge should be taken into consideration when caring for these children postoperatively.

### Acute management of acquired diaphragmatic hernia

Acute surgical intervention is warranted in all cases with acute obstructive symptomatic. After adequate fluid resuscitation, the patients are brought to theatre for emergency laparotomy. The previous subcostal incision may be used, and possibly extended cephalad up to the xiphoid for adequate exposure. The diaphragmatic breech is then identified. If the herniated content is incarcerated and oedematous, an extension of the defect may be required to safely reduce the bowel loops without causing further injury. In our experience the defect can then be closed with a primary repair using interrupted non-absorbable sutures The use of a synthetic mesh should be considered in isolated cases of large defects that would otherwise imply a non-tension-free repair.

Examination of the previously incarcerated loops should guide the possible need for resection in case of persistent ischemia.

## Conclusion

The use of split liver and living donor transplants is growing, especially for very young children, with specialized pediatric liver transplant programs yielding favorable outcomes due to their expertise. However, post-operative complications can arise after referral of patients to their local center of care, as seen in our three patients.

Based on our experience, any signs of bowel obstruction or respiratory distress in pLDLT recipients should raise strong suspicion for DH, especially within the first year post-transplant. Cross-sectional imaging plays a critical role in confirming the diagnosis and guiding the appropriate surgical strategy. When ischemic bowel is involved, careful handling is essential to prevent perforation. Extending the diaphragmatic defect may help minimize the risk of intraoperative injury, reducing the likelihood of postoperative complications and intra-abdominal infections—both of which could compromise the already fragile state of immunocompromised children recovering from liver transplantation.

Further studies are required to identify the proper pathophysiologic mechanisms of these iatrogenic hernias and prevent their occurrence in the postoperative period.

## Previous communication

This paper is not based on a previous communication to a society or meeting.

## Data Availability

The original contributions presented in the study are included in the article/Supplementary Material, further inquiries can be directed to the corresponding authors.
